# P2X7 Receptors Amplify CNS Damage in Neurodegenerative Diseases

**DOI:** 10.3390/ijms21175996

**Published:** 2020-08-20

**Authors:** Peter Illes

**Affiliations:** 1 Rudolf Boehm Institute for Pharmacology and Toxicology, University of Leipzig, 04107 Leipzig, Germany; peter.illes@medizin.uni-leipzig.de; Tel.: +49-341-9724614; 2 International Collaborative Centre on Big Science Plan for Purinergic Signalling, Chengdu University of Traditional Chinese Medicine, Chengdu 610075, China

**Keywords:** P2X7 receptor, neuroinflammation, neurodegenerative diseases, mechanical injury, ischemia, epilepsy, neuropathic pain, Alzheimer’s disease, Parkinson’s disease, multiple sclerosis, amyotrophic lateral sclerosis

## Abstract

ATP is a (co)transmitter and signaling molecule in the CNS. It acts at a multitude of ligand-gated cationic channels termed P2X to induce rapid depolarization of the cell membrane. Within this receptor-channel family, the P2X7 receptor (R) allows the transmembrane fluxes of Na^+^, Ca^2+^, and K^+^, but also allows the slow permeation of larger organic molecules. This is supposed to cause necrosis by excessive Ca^2+^ influx, as well as depletion of intracellular ions and metabolites. Cell death may also occur by apoptosis due to the activation of the caspase enzymatic cascade. Because P2X7Rs are localized in the CNS preferentially on microglia, but also at a lower density on neuroglia (astrocytes, oligodendrocytes) the stimulation of this receptor leads to the release of neurodegeneration-inducing bioactive molecules such as pro-inflammatory cytokines, chemokines, proteases, reactive oxygen and nitrogen molecules, and the excitotoxic glutamate/ATP. Various neurodegenerative reactions of the brain/spinal cord following acute harmful events (mechanical CNS damage, ischemia, status epilepticus) or chronic neurodegenerative diseases (neuropathic pain, Alzheimer’s disease, Parkinson’s disease, multiple sclerosis, amyotrophic lateral sclerosis) lead to a massive release of ATP via the leaky plasma membrane of neural tissue. This causes cellular damage superimposed on the original consequences of neurodegeneration. Hence, blood-brain-barrier permeable pharmacological antagonists of P2X7Rs with excellent bioavailability are possible therapeutic agents for these diseases. The aim of this review article is to summarize our present state of knowledge on the involvement of P2X7R-mediated events in neurodegenerative illnesses endangering especially the life quality and duration of the aged human population.

## 1. Introduction

ATP, originally thought to be solely the universal energy currency of cells, was discovered and characterized by Geoffrey Burnstock [1], as an extracellular non-adrenergic, non-cholinergic (NANC) neurotransmitter in smooth muscle organs such as the taenia coli. In the following decades Burnstock gradually developed his hypotheses to describe many facets of his holistic theory on a “purinergic signaling system”. Soon after recognizing NANC neurotransmission, he conceptualized the idea of “co-transmission” to depict the co-storage and co-release of ATP with other classic neurotransmitters [2]. Subsequently, the concept of co-transmission flourished, and a wealth of data demonstrated that neurons both in the peripheral and central nervous system may release more than one transmitter [3]. In addition to its transmitter function in the nervous system, purine and pyrimidine nucleotides were also recognized to act as signaling molecules coordinating the function of almost every cell in the animal/human organism. Receptors (Rs) which are targets for these nucleotides have been classified into two types, the ligand-gated cationic channels P2X (seven mammalian subtypes: P2X1-7) and the G protein-coupled P2YRs (eight mammalian subtypes: P2Y1, 2, 4, 6, 11–14) [4,5,6]. The enzymatic degradation product of ATP, adenosine may act at four types of receptors, termed A1, A2A, A2B and A3 [7].

Geoffrey Burnstock’s primary interest in the last period of his life was in neurodegeneration and -regeneration. He wrote many conceptual review articles on the involvement of ATP in brain injury, stroke, ischemia, epilepsy, chronic pain, Alzheimer’s disease, Parkinson’s disease, multiple sclerosis and amyotrophic lateral sclerosis [8,9]. The aim of the present review is to summarize the state of our present knowledge on a special subtype of P2XRs, the P2X7R, in the amplification of CNS damage during neurodegeneration and the elucidation of P2X7R antagonists as possible pharmacological means to alleviate the deleterious consequences of this group of illnesses for mankind.

## 2. Purinergic P2X7 Receptors

Ionotropic P2X7Rs are members of the P2X purinoceptor family, which were cloned and characterized in 1996 [10,11]. P2X7Rs, similar to other P2XR-types form trimeric assemblies of three identical subunits [12,13]. Each subunit has N- and C-terminal regions, two transmembrane areas, and a large extracellular loop; the agonist-binding pouch of the receptor is located at the intersection of two neighboring subunits [14,15]. Several P2X7R isoforms derived from alternative splicing were identified both in humans [16], and in rodents [17,18]. Some variants are expressed and functional, e.g., human P2X7A and P2X7B, as well as mouse and rat P2X7 variant K. Interestingly, the co-expression of the two splice variants P2X7A and P2X7B results in a single receptor with enhanced function [19].

Three properties of the P2X7R are distinguishing characteristics: Firstly, it is activated by high concentrations of ATP in the millimolar range, clearly surmounting concentrations needed to activate other P2XRs, which are stimulated by ATP concentrations in the micromolar range [10]. Secondly, it is an ATP-gated non-selective cationic channel, allowing the passage of Na^+^, K^+^, and Ca^2+^ through the cell membrane leading to inward current/depolarization at the resting membrane potential. However, similar to P2X2 or P2X4Rs, long-lasting occupation of the agonist-binding pouch of the P2X7R was thought for a couple of years to result in a time-dependent dilation of the channel to constitute a pore, through which molecules previously not permeating the channel may pass into either direction ([20,21,22]; but see below). Thirdly, the C-terminus of P2X7Rs is much longer than that of other P2XRs and has been implicated in regulating receptor function, including signaling pathways, cellular localization, protein-protein interactions, and post-translational modification [18,23,24].

With respect to P2X7R dilation it was shown later that the interpretation of whole cell recording patch-clamp data obtained by reversal potential measurements were probably misleading [25]. Participation of associated channel-forming proteins has been implicated (e.g., Pannexin-1 [Panx-1]), but convincing evidence now supports the view that the P2X7R by itself is endowed with the ability to conduct large organic cations [26], or form a large conductance pore [22,27].

**Figure 1:**
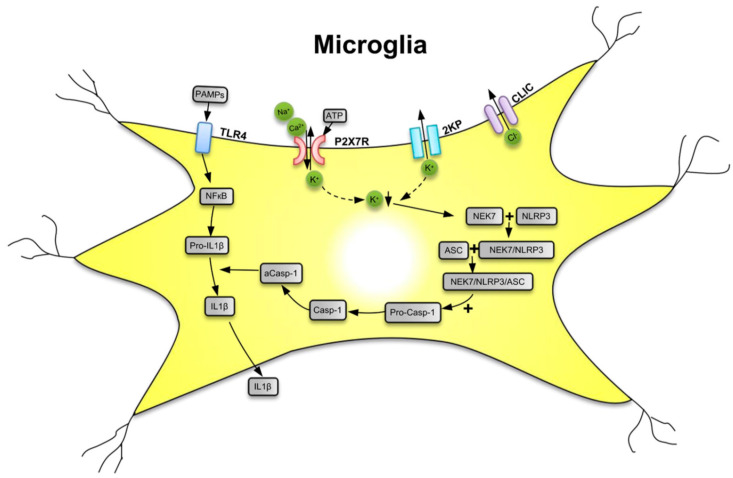
Secretion of interleukin-1β (IL-1β) from microglial cells via involvement of the nucleotide-binding, leucine-rich repeat, pyrin domain containing 3 (NLRP3) inflammasome. Pathogen-associated molecular patterns (PAMPs; e.g., bacterial lipopolysaccharide (LPS)) act on toll-like receptor-4 (TLR4) and cause its phosphorylation. In consequence, in the cell nucleus, NF-κB is activated, which promotes the synthesis of the NLRP3 inflammasome and pro-IL-1β, both accumulating in the cytosol in their inactive forms. The activation of NLRP3 is primarily due to a decrease of the intracellular K^+^ concentration [K^+^]_i_, initiated by the stimulation of P2X7Rs by high local concentrations of the molecule ATP, which is considered to be a danger-associated molecular pattern (DAMP). P2X7Rs allow the inward flux of Na^+^/Ca^2+^ and in exchange the outward flux of K^+^, leading to a fall in [K^+^]_i_. The opening of two-pore domain potassium channels (2KP) may also lead to an impoverishment of cytoplasmic K^+^. A further stimulus for NLRP3 activation is the outward flux of Cl^−^ through chloride intracellular channels (CLICs). TLR4, P2X7Rs, 2 KP channels, and CLICs are all located in the cell membrane of microglia. A sensor for the fall in [K^+^]_i_ is the NEC7 serine/threonine kinase. NEC7 is able to form a complex with NLRP3, which is still inactive, but after constitution of a still larger multimeric complex with apoptosis-associated speck-like protein (ASC) binds also pro-caspase-1 (pro-Casp-1). In consequence, pro-Casp-1 is cleaved to Casp-1, which then by its activated form aCasp-1 degrades pro-IL-1β to mature IL-1β. Then, IL-1β leaves the cell by a number of mechanisms to the extracellular space and exerts its effects as a neuroinflammatory cytokine. K^+^ with downward arrow, decrease of the K^+^ concentration. Reproduced with permission from [37].

The P2X7R is a major driver of inflammation; it is widely expressed by cells of the innate and adaptive immune systems all over the animal/human organism [18,28]. The preferential location of P2X7Rs in the CNS is on microglia, the resident macrophages of the brain [29]. However, although at lower density, these receptors are also expressed at neuroglial cells (e.g., astrocytes and oligodendrocytes) [30,31,32]. Microglia are equipped with a battery of pattern recognition receptors that stereotypically detect pathogen-associated molecules (PAMPs) such as lipopolysaccharide (LPS) from bacterial infection or danger-associated molecular patterns (DAMPs) such as ATP [33,34,35] (Figure 1). Both PAMPs and DAMPs are needed for the microglial release of the cytokine interleukin-1β (IL-1β) which occurs in two consecutive steps. In a first step, stimulation by LPS of toll-like receptor 4 (TLR4) leads to accumulation of cytoplasmic pro-IL-1β, and then, in a second step, the ATP-dependent stimulation of P2X7R, and the subsequent activation of nucleotide-binding leucine-rich repeat, pyrin domain containing 3 (NLRP3) inflammasome occurs. NLRP3 stimulation initiates the cleavage of pro-caspase-1 to caspase-1 and the subsequent enzymatic degradation of pro-IL-1β to the mature IL-1β, by caspase-1-induced proteolysis. Although P2X7Rs are present at astrocytes, the lack of NLRP3 leads to their inability to release IL-1β [36].

Microglia exist in a highly ramified form under resting conditions but get activated by changes in brain homeostasis [38]. This leads through different intermediary activation states to polarization into an amoeboid form which can phagocytose pathogenic bacteria and release a number of bioactive molecules such as the pro-inflammatory cytokines IL-1β, IL-6, IL-18, tumor necrosis factor-α (TNF-α), chemokines, proteases, reactive oxygen/nitrogen species, and probably also the excitotoxic ATP and glutamate by vesicular exocytosis [3,34,39]. In addition to this classically activated M1 microglial/macrophage phenotype, typically releasing the mentioned destructive pro-inflammatory mediators, the alternatively activated M2-phenotype clears cellular debris through phagocytosis and releases numerous protective factors (IL-4, IL-13, nerve growth factor [NGF], and fibroblast growth factor [FGF]) [40,41]. Recent evidence indicates that the M1/M2 dichotomy is an oversimplified conceptual framework; nonetheless it is of great significance for understanding the outcome of neurodegenerative illnesses. While M1 microglia are involved in cell damaging processes superimposed on the basic pathogenic mechanism in e.g., Alzheimer’s disease or Parkinson’s disease, M2 microglia are involved in the accompanying neuro-regeneration [42,43,44].

P2X7R bearing cells in the CNS may undergo necrosis. With sustained agonist stimulation, pore-opening allows excessive Ca^2+^ influx, depletion of intracellular ions as well as metabolites, and ultimately cell death. This has led originally to the conceptualization of a dogma that pore-formation is cytolitic, with description of P2X7R as a “death/suicide” receptor [10,45]. In addition to this immediate reaction, apoptotic cell death due to the activation of the caspase cascade via P2X7R-dependent stimulation of the NLRP3 inflammasome was also recognized [46,47]. Somewhat later it was appreciated that in contrast to the long-held view of cell death, P2X7Rs may also promote proliferation in microglia [48,49] and tumor cells [50]. Interestingly, this effect was associated with the so-called pore-dilation of the receptors, because a point mutant of a P2X7R with intact channel function but ablated pore-forming capacity could not establish microglial proliferation [49].

Although it was absolutely clear that P2X7Rs are localized at the highest density on microglia and their presence on neuroglial cells is much less pronounced, there has been a long-lasting discussion whether there are neuronal P2X7Rs at all (for a summary of the opposing views see [51,52]). The main arguments for the absence of P2X7Rs at neuronal somata and terminals are the following: (1) Three classes of P2X7R antibodies, binding to the C-terminal or ectodomain epitopes, yielded different immunoreactivity (IR) patterns [53]. Hence any evidence for neuronal P2X7R-labelling by antibodies was considered to be questionable; (2) All antibodies stained not only hippocampal structures of wild-type (wt) mice, but also those of two separate P2X7R knockout strains generated by the pharmacological companies Pfizer or Glaxo-Smith-Kline [54]. This was due to the fact that in these “classic” P2X7-deleted mice, some immunohistochemically/functionally active splice variants evaded the knockout strategy (see Section 12). By means of a novel, transgenic Tg(P2X7-EGFP) mouse which allowed visualization of EGFP-tagged P2X7Rs, no protein expression was found on neurons in the CNS [32]; (3) Electrophysiological experiments on neurons in hippocampal mixed neuron-astrocyte cultures, hippocampal brain slices, and spinal cord slices, failed to supply evidence for functional neuronal P2X7Rs, although these receptors were present at neighboring astrocytes [55,56,57]. (4) P2X7Rs present in microglia and neuroglia are perfectly capable to execute all neuronal effects. There is a plethora of data collected by a diversity of methods showing that microglia modulate neuronal functions by the release of biologically active cell products (cytokines, chemokines, proteases, reactive oxygen and nitrogen species, and even glutamate and ATP) [34]. Astrocytes also profoundly interact with neurons by both exocytotically released gliotransmitters (glutamate, ATP, GABA, D-serin, taurine) and further signaling molecules entering the extracellular space through channels/transporters (e.g., ATP via connexin hemichannels, pannexin channels, maxi anion channels, volume-regulated anion channels, the Ca^2+^-dependent Cl^−^ channel bestrophin, the calcium homeostasis modulator 1 (CALHM1-3) and even by the P2X7R channel itself) [58].

After having clarified these issues related to P2X7Rs, we turn our attention to the individual neurodegenerative diseases and discuss them as follows.

## 3. Mechanical Damage to the CNS

Weight drop injury to the thoracic spinal cord T12 segment of rats in vivo caused massive release of ATP from damaged cells as visualized by a bioluminescence method [59]. This excessive release of ATP led in spinal cord neurons to high frequency spiking, irreversible increases of intracellular Ca^2+^ [Ca^2+^]_i_ and apoptotic cell death. Numerous terminal deoxynucleotidyl transferase dUTP nick end labeling (TUNEL)-immunoreactive (IR) cells, exhibiting also nuclear chromatin condensation and fragmentation, indicated apoptosis. Many cells showed strong staining for P2X7R-IR. Eventually, injection of the moderately selective P2X7R antagonist oxidized ATP (oxATP) rostral and caudal from the epicenter of the injury caused a more rapid functional recovery than in the vehicle treated controls. In a similar experimental arrangement, the more selective and reversible P2X7R antagonist Brilliant Blue G (BBG) also reduced spinal cord anatomic damage and improved motor recovery [60,61]. Further, BBG treatment not only protected spinal cord neurons from purinergic defects but also reduced local inflammatory responses (extent of microglial and astrocytic activation, number of infiltrating neutrophils). It was concluded that spinal cord injury induces the release of high amounts of the excitotoxic ATP, the subsequent long-lasting activation of P2X7Rs and the resulting death of motoneurons in the peritraumatic zone. These effects were terminated by the compensatory up-regulation of the ecto-nucleoside triphosphate diphosphohydrolases (NPDases) degrading ATP to its metabolite AMP [62,63], which in consequence more rapidly terminated P2X7R activity [64].

These findings were confirmed for the rat spinal cord [65] and extended for the rat parietal cortex [66]. Whereas in the former study BBG was used as a P2X7R antagonist, in the latter study the P2X7R antagonist A-804598 of higher selectivity was utilized. In another model of mechanical injury of high pathophysiological significance, damage to the retina was induced by generating elevated pressure in the anterior chamber of the eye [67]. Such an increase in intraocular pressure caused damage to the optic nerve, formed by the axons of retinal ganglion cells, resulting in their retrograde death and consequent loss of vision. In humans, glaucoma is a frequent cause of blinding especially in older ages. Elevation of intraocular pressure increased mRNA for inflammasome components in rat and mouse retinas. Under in vitro conditions stretch or swelling of astrocytes from optic nerve head demonstrated increased expression of the pro-inflammatory cytokine IL-1β, an effect that was eliminated by the degradation of extracellular ATP by apyrase, an ecto-ATPase enzyme, or P2X7R antagonistic substances such as BBG and A-839977 [68]. Axotomy of the mice optic nerve caused a delayed loss of retinal ganglion cells and the activation of phagocytic microglia, reactions which could be also blocked by P2X7R antagonists.

## 4. Ischemic Damage to the CNS

It was shown around the turn of the century that permanent focal cerebral ischemia (medial cerebral artery occlusion; MCAO) in rats up-regulated the P2X7R-IR in the cortical peri-infarct (penumbral) region of spontaneously hypertensive rats [69]. This up-regulation was time-dependent in that P2X7R-IR was increased post-ischemically on GSA-B4 positive microglial cells (after one to four days), on glial fibrillary acidic protein (GFAP) positive astrocytes (after four days) and on tubulin βIII positive neurons (after four to seven days). Thus, the earliest P2X7R expression occurred on microglia, which established the first line of immune defense in the brain, followed by up-regulation of IR on astrocytes and neurons. Western blot analysis of the cortical tissue around the area of necrosis/apoptosis indicated the increase of the P2X7R protein. Detailed investigations of the same group of authors demonstrated that a P2R-type probably of the P2X7R-subtype is responsible for the partly reversible damage to neurons in the penumbra after MCAO [70]. The intracerebroventricular (i.c.v.) application of the non-selective P2R antagonist PPADS reduced the infarct volume and decreased the number of irreversibly injured cells in the brain of spontaneously hypertensive rats. Most interestingly the PPADS-treated group of animals showed a reduction of paresis-induced side slips compared with the ACSF-treated group in a behavioral paradigm. These data were confirmed by using BBG instead of the non-selective PPADS, to demonstrate that selective blockade of P2X7Rs greatly reduced the extent of brain damage when compared with the treatment of vehicle alone [71].

Transient global cerebral ischemia induced by occlusion of the bilateral common carotid arteries and both vertebral arteries caused a still more pronounced damage to the brain than MCAO [72,73]. In this case, P2X7R-IR was up-regulated in the CA1 area of the rat hippocampus, accompanied by shedding of microvesicle-like components and higher IL-1β expression, most probably favoring delayed neuronal death because of neuroinflammation. This resulted also in learning memory deficits as determined in the Morris water maze [72]. I.c.v. infusion of a whole range of P2X7R antagonists (BBG, oxATP, A-438079) significantly increased the survival rate of the animals, reduced the learning memory deficits, glial activation and inflammatory cytokine overexpression in the hippocampus.

The role of P2X7Rs in ischemic/reperfusion injury is generally considered as deleterious, and accordingly blockade of this receptor-type is expected to be neuroprotective [74]. In fact, in primary cortical neurons and cortical brain slices, oxygen-glucose deprivation (OGD) generated a post-anoxic inward current accompanied by massive Ca^2+^ influx and in consequence neuronal death [71] (but see also [75]). BBG delayed/reduced the post-anoxic current and prevented neuronal damage under in vitro conditions. In accordance with the early appearance of P2X7R-IR on microglia after MCAO [69,76] it was concluded that activated microglia, by its well-known detrimental properties boosts the neuronal injury caused by ischemia itself (see Section 2). Hence, Reactive Blue 2, a non-selective P2R antagonist improved sensorimotor deficit and restricted the volume of infarction after MCAO [76].

By contrast, an indirect, but beneficial effect of P2X7Rs was demonstrated in BV2 microglial cultures, in which OGD promoted cell death [77]. Less microglial cells would of course mean lower vulnerability of neurons to ischemia/reperfusion injury. Whereas the increased release of the excitotoxic neuro/gliotransmitter glutamate by P2X7R activation was supposed to add up to neuronal injury during ischemia [78], the increased release of the inhibitory neuro/gliotransmitter GABA shown under similar conditions may have the opposite effect [79]. Thus, P2X7Rs were reported both to exert neurodegenerative and neuroprotective effects during an ischemic insult, admittedly with the first type of reactions predominating. A solution to this riddle may be among others the contrasting functions of the M1/M2 microglial phenotypes (see Section 2).

Preconditioning, using a preceding sublethal ischemic insult is an attractive strategy for protecting neurons from subsequent ischemic damage [80,81]. A 15-min transient MCAO caused ischemic tolerance due to the long-lasting up-regulation of hypoxia-inducible factor 1-α (HIF-1α) in astrocytes. The selective blockade of astrocytic metabolism by the astrocyte selective toxin fluorocitrate or their P2X7R-mediated HIF-1α overproduction, both abolished ischemic tolerance. Interestingly, not only preconditioning but also postconditioning is able to decrease the severity of the ischemic damage [82]. The postconditioning protocol means that after bilateral carotid artery occlusion for 12 min, intermittent short-lasting carotid artery occlusions are applied, limiting the extent of the cerebral infarct size as well as the extent of the memory and motor coordination deficits. This postconditioning procedure also depended on P2X7Rs, as demonstrated by its abolition by pre-treatment with intraperitoneal (i.p.) BBG.

As mentioned earlier, the pharmacological blockade of P2X7Rs ameliorates the OGD-induced post-anoxic inward current in cortical neurons in culture and the subsequent cell death [71]. In this case two alternative mechanisms of action of P2X7R antagonists are conceivable: (1) these substances may inhibit the release of the excitotoxic ATP/glutamate via P2X7Rs and/or Panx-1 channels; and (2) they may inhibit the neurodegeneration caused by P2X7Rs located at astrocytes/microglia. The first mode of action is favored by experiments showing that both P2X7R and Panx-1 blockers counteracted the deleterious effect of OGD in acutely prepared cortical brain slices, cortical neuronal cultures and organotypic cultures of this area of the brain [83]. Patch-clamp measurements confirmed that in all three types of preparations OGD induced a post-anoxic inward current sensitive to blockade of P2X7Rs by A-438079, and Panx-1 channels by probenecid. Although hypoxia/ischemia was shown to open both connexin hemichannels and pannexin channels in the CNS [84], post-anoxic depolarization of hippocampal or cortical pyramidal neurons are devoid of connexin hemichannels [85] and must be due to permeation through the pannexin channels [83].

By contrast to this conclusion, in CA1 hippocampal pyramidal cells Madry et al. [86] could not confirm the involvement of Panx-1 channels in post-anoxic depolarization; this response was resistant to blockade by carbenoxolone, lanthanum and mefloquine, three standard (hemi)channel inhibitors, but was sensitive to AP-5, NBQX and bicuculline, inhibitors of NMDA, AMPA and GABA_A_Rs, respectively. In partial agreement with these results, in rat hippocampal astrocytes the post-anoxic depolarization was inhibited by the NMDA-R antagonist AP-5, but not by the wide-spectrum P2R antagonist PPADS [75].

## 5. Epilepsy

The involvement of P2X7Rs in the generation/maintenance of epileptic seizures, especially in their most severe form of a status epilepticus (SE), have attracted a lot of interest with an accordingly high number of publications available. Unfortunately, this also resulted in the production of sometimes controversial data and conclusions, which are not quite easy to sort out in the present review. The confusion is due to various factors, in the first line to the inhomogeneity of the clinical/experimental state (primary or secondary generalized seizures), the various animal models used (chemically- or electrically-induced epilepsy), the temporal parameters (acute or chronic epileptic seizures), and the end-points measured (EEG signals or tonic/clonic muscular cramps).

The original finding, that SE induces an up-regulation of P2X7R-IR in epilepsy-relevant areas of the brain (hippocampus, neocortex), was observed repeatedly both in human and rodent tissues (e.g., [87,88,89]. In consequence, it was logical to suggest that damage to neurons by pathological, high frequency firing during epileptic fits may cause a massive release of ATP and the consecutive activation of P2X7Rs located in the first line at microglial cells; this is expected to release bioactive, detrimental molecules (see Section 2). The resulting brain inflammation appears to play a key role in the generation of seizures and the pathogenesis of epilepsy [90,91].

NTPDase1 hydrolyses nucleoside 5′-triphosphates and -diphosphates equally well and is the main ATP degrading enzyme in microglia [92]. NTPDase1-deficient (CD39^−/−^) mice, due to less enzymatic degradation of ATP, exhibited increased levels of ATP and decreased levels of adenosine in their cerebrospinal fluid [93]. In addition, they were prone to handling-induced and spontaneous epileptic seizures [93]. HPLC measurements of ATP and its metabolites (ADP, AMP, adenosine) only partially supported this hypothesis, because during pilocarpine-induced temporal lobe epilepsy the extracellular ATP concentrations showed only a tendency to be increased in the microdialysate of the hippocampus [94]. This, however, may be explained by the poor sensitivity and temporal resolution of the HPLC measurement technique [95].

To find out whether P2X7R occupation is causally involved in SE, kainic acid was injected into the basolateral amygdala of Tg(P2X7-EGFP) mice, in order to induce EEG seizure activity [88,96]. This method was considered to be superior to systemic application of kainic acid, because of the immediate availability of the convulsant in an epilepsy-relevant brain structure. The transgenic mice expressing enhanced green fluorescent protein (EGFP) under the control of the P2X7 promoter made it possible to follow the expression of the receptor protein by immunohistochemical methods. This was necessary, because P2X7R antibodies were found to be quite unreliable; different charges binding to different epitopes yielded variable receptor distribution in the CNS (see Section 2).

Jimenez-Pacheco et al. [96] concluded that the intra-amygdala injection of kainic acid and the ensuing EEG seizure activity led to a selective increase of the receptor in CA1 pyramidal cells and granule neurons as well as microglia of Tg(P2X7-EGFP) mice. They identified the EGFP-positive cells by co-labelling with the neuron-specific antibody NeuN and the microglia-specific antibody Iba1, respectively, and also demonstrated larger Bz-ATP-induced currents in supposed neurons of hippocampal brain slices prepared from epileptic, in comparison with non-epileptic mice. However, the NeuN co-expression with EGFP was shown only with the low-resolution fluorescence microscopy rather than with the high resolution confocal laser scanning microscopy; counter-staining with the astrocytic marker GFAP as a negative control was also missing. Moreover, the authors failed to electrophysiologically discriminate neurons from astrocytes by injecting depolarizing current into their EGFP-positive cells and looking for spiking (neurons) or non-spiking (astrocytes). Hence, their arguments for a selective up-regulation of P2X7Rs in neurons are not really convincing.

Another issue of uncertainty may be inherent to the animal model used. Tg(P2X7-EGFP) mice certainly do not allow to unequivocally identify the P2X7R protein. The BAC RP23-181F3, which has been utilized to generate the P2X7-EGFP reporter line, encompasses the entire P2X4 gene [97]. P2X4Rs were shown to be up-regulated in the hippocampus of mice who underwent kainic acid-induced SE [98]. Further, systemic application of three blood-brain barrier permeable P2X7R antagonists (BBG, JNJ-47965567, AFC-5128) to wt mice failed to alter the maximal electroshock and pentylenetetrazole seizure thresholds, although BBG exhibited a moderate retarding effect, and JNJ-47965567 and AFC-5128 showed long-lasting delay in kindling development [99]. It is also interesting to mention that P2X7^−/−^ mice showed greater susceptibility to seizures induced by pilocarpine, in contrast to picrotoxine, than their wt littermates [100]. Both administration of P2X7R antagonists and gene silencing of this receptor increased pilocarpine-induced seizure susceptibility. Thus, the effects of P2X7R antagonists depended on the type of mice and the type of the convulsant drug used.

However, in spite of these uncertainties, it is believed by most authors that P2X7R antagonists are potential drugs to prevent epilepsy as based on experiments carried out both in mouse [101,102], and rat animal models [103].

Although it has been concluded that the deleterious changes by SE primarily arise via neuronal targets, more recently an astrocytic mode of action has been considered to be of comparable importance [104,105]. Neural stem cells evolved as an additional/alternative player in pathological circuitries shaped by SE; systemic kainic acid- or pilocarpine-induced SE potentiated Bz-ATP currents in astrocyte-like neural progenitor cells (NPCs) of the subgranular zone of Tg(nestin-EGFP) mice [106,107]. It was shown that in hippocampal brain slices, pathological firing in 4-aminopyridine-treated dentate gyrus granule cells elicited increased Bz-ATP currents of the underlying subgranular zone NPCs [108]. SE may foster the proliferation of NPCs and subsequently these NPCs may migrate into ectopic locations of the hippocampus, differentiate into mature neurons and become integrated into aberrant neuronal circuits resulting in the manifestation of chronic epilepsy [107]. The activation of P2X7Rs by ATP causes necrosis/apoptosis of NPCs and thereby may prevent these harmful processes [106,109].

## 6. Chronic Pain

Chronic pain includes the inflammatory, neuropathic and cancer pain states. In all these cases there is a primary or secondary strong inflammatory component, in the case of neuropathic pain, restricted to peripheral or central neuronal tissues. Neuropathic pain has the cardinal symptoms of spontaneous, continuous or paroxysmal pain, hypersensitivity to painful stimuli (lower threshold) and allodynia. Because of the inflammatory component, all types of chronic pain states were shown to respond to the pharmacological blockade of P2X7Rs [105,110,111,112]. In the following, we will discuss in the first line neuropathic pain, but will also refer to P2X7R involvement in inflammatory pain which may include also destruction of small peripheral C-fiber terminals.

Constriction injury to the L5 lumbar nerve, as a model of neuropathic pain, activated microglial P2X4Rs in the dorsal horn of the spinal cord and lead via the release of brain-derived neurotropic factor (BDNF) to hyperexcitability of dorsal horn lamina I neurons [113,114]. However, simultaneously to the publication of these findings, antagonists and knockout mice have implicated microglial P2X7Rs in chronic neuropathic (and inflammatory) pain through the release of IL-1β from microglia/macrophages [105,115,116]. Peripheral nerve injury (transection of the tibial and common peroneal nerves) caused an increase of both P2X7R mRNA and protein in the spinal cord [117]. Double labelling immunohistochemistry demonstrated that cells expressing P2X7R protein after nerve injury were predominantly microglia. The intrathecal administration of the P2X7R antagonist A-438079 suppressed the development of mechanical hypersensitivity in the hindpaw, ipsilateral to nerve damage.

With respect to the transduction mechanism of P2X7Rs, IL-1β was found to be secreted by spinal cord microglia corresponding to the “two-hit hypothesis” [118]. LPS occupied TLR4, leading to the accumulation of cytoplasmic pro-IL-1β, and after that ATP stimulated P2X7Rs, promoting NLRP3 inflammasome-mediated caspase-1 activation, which degraded pro-IL-1β to mature IL-1β becoming thereafter secreted from microglial cells ([119]; see Section 2). In fact, in spinal cord slices of wt mice, LPS induced the release of IL-1β in a manner antagonized by the selective P2X7R blocker A-438079, while LPS was inactive in spinal cord slices taken from P2X7 knockout mice [118]. In behavioral studies intrathecal injection of LPS to the lumbar spinal cord produced mechanical hyperalgesia in rat hindpaws, which was attenuated by concomitant injections of either oxATP or A-438079.

Further arguments for the involvement of IL-1β in (inflammatory) pain was supplied by experiments showing that i.p. administration of the P2X7R antagonist A-839977 reduced thermal hyperalgesia produced by intraplantar application of Complete Freund’s Adjuvant (CFA), known to induce long-lasting inflammation [120]. A-839977 produced robust antihyperalgesia in wt mice, but this effect was completely absent in IL-1αβ knockout animals. IL-1β is released from microglia, packed in extracellular vesicles of variable shape/size generated by the outward blebbing of the microglial plasma membrane; P2X7R activation was found to be the initiating factor for blebbing [121,122]. After spinal nerve ligation in rats, the number of extracellular vesicles was increased in the cerebrospinal fluid and dorsal horn of the spinal cord [123]. Intrathecal injection of extracellular vesicles caused a marked decrease of paw withdrawal threshold to painful pressure and paw withdrawal latency to thermal pain, suggesting that such vesicles contain a pro-inflammatory substance, most likely IL-1β. Other pro-inflammatory cytokines such as TNF-α were also released from microglia in consequence to P2X7R stimulation and were shown to participate in allodynia/hyperalgesia following trigeminal nerve injury [124].

It was also reported that activated P2X7Rs may generate in microglia/macrophages reactive oxygen species (ROS). In cultured spinal cord dorsal horn neurons, the ATP- or Bz-ATP-induced ROS production was eliminated both by the ROS scavenger *N*-tert-butyl-α-phenylnitrone and the P2X7R antagonist A-438079 [125]. Intrathecal application of Bz-ATP induced robust biphasic spontaneous nociceptive behavior. Pre-treatment with A-438079 abolished both the first and second phase of this response, while ROS scavengers attenuated only the second phase.

In addition to mechanical damage to major peripheral nerves also diabetes mellitus (DM) is a frequent cause of neuropathic pain. Streptozocin alone was injected to destroy pancreatic β-cells in order to generate a model of DM type I [126]; alternatively, after streptozocin injection, animals were fed with a high-sugar and high-fat diet for 4 weeks to generate a model of DM type II [127]. Streptozocin-treated mice expressed increased P2X7R protein in the dorsal horn of the lumbar spinal cord, and P2X7R-IR was co-localized with the microglial marker Iba1 [126]. When these mice were injected intrathecally with the P2X7R antagonist A-740003, or when P2X7R^−/−^ mice were rendered diabetic/neuropathic, an attenuated progression of mechanical allodynia was observed. In models of Type II DM, long non-protein-coding (Lnc) RNAs were increased in the DRGs of rats, and a small interference (si) RNA generated against the LncRNAs counteracted the previously increased mechanical withdrawal threshold and thermal withdrawal latency [127]. In parallel to the functional changes, the increased P2X7R mRNA and protein in the DRGs of diabetic rats were also reduced by the siRNA treatment.

In accordance with the preferential localization of P2X7Rs at microglia/macrophages, all previously discussed experiments strongly support the notion that such receptors are intimately involved in pain states. However, P2X7Rs, although at lower density, are also present on neuroglial cells [31]. It has been reported that satellite glial cells in DRGs intimately communicate with the neighboring sensory neurons via ATP release and P2X7Rs. The release of ATP from electrically stimulated cultured DRG neurons has been confirmed by sniffer patch recordings [128]. FM1-43 photoconversion analysis and the Ca^2+^-dependency of the release process indicated its exocytotic nature. A second publication by this group of authors reported that P2X7Rs in satellite cells tonically inhibited the expression of P2X3Rs in neurons [129]. Reducing P2X7R expression by siRNA or blocking P2X7Rs by pharmacological antagonists elicited P2X3R upregulation, increased the activity of sensory neurons responding to painful stimuli and evoked increased α,β-methylene ATP (α,β-meATP)-induced flinching as a measure of pain. Although α,β-meATP-induced flinching is a model of acute pain, further experiments in rats pre-treated with CFA also suggested that in this model of chronic inflammatory pain, P2X7Rs exert an inhibitory tone on P2X3R-mediated analgesia.

In conclusion, both spinal microglia and DRG satellite glial cells are endowed with P2X7Rs and appear to participate in the modulation of chronic pain. Tetanic stimulation to the sciatic nerve in vivo produced long-lasting hyperalgesia and allodynia in rats [130]. This pain reaction depended on the undisturbed functioning of astrocytes, because fluorocitrate partially impeded the tetanic stimulation-induced reduction of the paw withdrawal latency. Electrophysiological studies in spinal cord slices also showed that long-term potentiation (LTP) of C-fiber/lamina I neuron synapses is induced by tetanic stimulation to the left sciatic nerve [131]. The P2X7R antagonists oxATP and BBG prevented the initiation of spinal LTP both in vivo and in spinal cord slices in vitro, and alleviated spinal analgesia [132]. Double immunofluorescence showed co-localization of P2X7R-IR with the microglial marker OX-42, but not with the astrocytic marker GFAP or the neuronal marker NeuN. Pre-administration of the IL-1R antagonist IL-1ra blocked the induction of spinal LTP. These findings as a whole show that repetitive activation of C-fiber inputs to the spinal cord dorsal horn induce a long-lasting potentiation of the sensory quality pain and that this process is probably depending on both astroglial and microglial P2X7Rs.

Tolerance is a well-known disadvantage of chronic morphine treatment. Various neuronal reasons have been proposed for this phenomenon, but more recently spinal microglial P2X7R up-regulation was added to the existing hypotheses [133]. After chronic exposure to morphine, the protein level of spinal P2X7Rs was up-regulated and intrathecal application of BBG attenuated the loss of morphine analgesic potency, P2X7R up-regulation, and microglial activation. BBG prevented the analgesic tolerance to morphine, but once it was established, it was no longer affected by BBG. Instead of BBG, also the intrathecal administration of a palmitoylated peptide corresponding to the Y_382-384_ site in the C-terminus of P2X7Rs suppressed microglial reactivity and preserved the antinociceptive effect of morphine [134]. The modulation of morphine-tolerance at neurons was established via the release of IL-18 from microglia, which probably acted at IL-18Rs at astrocytes to induce the release of the gliotransmitter D-serine [135].

The duration of experimental neuropathic pain was largely prolonged by morphine, due to the amplification of spinal NLRP3 inflammasome activation in microglia [136]. This paradoxical reaction depended on the release of IL-1β from microglia. In an elegant methodological approach Grace et al. [136] transfected into spinal microglia a novel designer receptor exclusively activated by designer drugs (DREADD) coupled to a G_i_ protein. After its activation by clozapine-*N*-oxide the morphine-induced persistent sensitization was prevented or enduringly reversed. It was further concluded that after peripheral nerve injury, morphine treatment results in persistent DAMP release via TLR4, P2X7R and caspase-1, which are involved in the formation/activation of NLRP3 inflammasomes [137].

The assessment of abnormal tactile hypersensitivity in mice of different genetic background after spared nerve injury showed robust inter-strain differences [138]. Using genome-wide linkage analyses, an association between mechanical allodynia and the Pro45Leu mutation of the mouse P2X7 gene was established; mouse-strains in which P2X7Rs have impaired pore formation as a result of this mutation showed less allodynia than mice with the pore-forming allele. A number of gain- and loss-of-function small nucleotide polymorphisms (SNPs) in patients with diabetic peripheral neuropathic pain was associated with higher pain intensity scores in females but not males [139].

## 7. Alzheimer’s Disease (AD)

Individuals with AD can be differentiated into three widely accepted subgroups: (1) AD with autosomal dominant inheritance; (2) Early-onset AD with genetic risk (<65 years); and (3) Late-onset AD which is by far the most common form with a polygenic causality [140,141,142]. The amyloid cascade hypothesis has dominated the strategy of AD research for decades, despite evidence for its limitations, including known heterogeneity of the dementia syndrome in the population and the narrow focus on a single molecule—the amyloid beta protein (Aβ) whose aggregates were supposed to be causal for AD-type dementia [143,144,145]. Aβ is a small molecule that is derived from the larger amyloid precursor protein (APP) after sequential cleavage by β- and γ-secretases, in contrast to the soluble fragment of APP (sAPPα) generated by α-secretase [146]. Extracellular Aβ aggregates are considered neurotoxic, while sAPPα appears to be neurotrophic and neuroprotective [145,147].

Another macromolecule tightly linked to AD development is the tau-protein, known for its function which consists of stabilizing the N-terminal part of microtubules in neuronal axons and astrocytes [145,148,149]. Aggregates of Aβ form amyloid plaques which when surrounded by neurites filled with fibrillary hyperphosphorylated tau, give rise to so-called neurofibrillary tangles considered as hallmarks of AD [140,150]. Autosomal dominant AD is associated with mutations in genes that encode proteins with a role in Aβ generation, including APP, as well as presenilin-1 and -2 (PSEN1, PSEN2) encoding the two proteins, respectively, which are involved in APP processing [151].

Although there is little correlation between the presence of amyloid plaques/neurofibrillary tangles with the degree of cognitive decline in AD [152], most of the available animal models of this disease are either transgenic mice obtained by random integration of gene variants that encode proteins implicated in AD pathology [140] or rodents injected with Aβ into their brains (see below).

**Figure 2:**
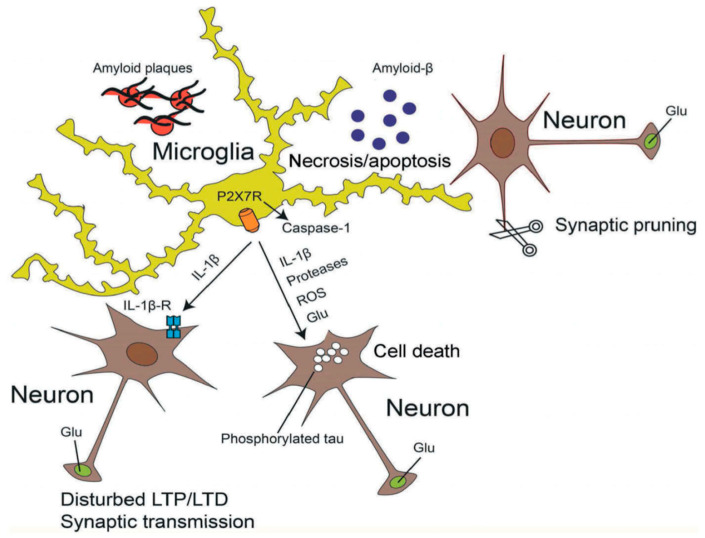
Effects of activated microglia on neighboring neurons. Soluble β-amyloid (Aβ) aggregates and thereby forms fibrillary plaques surrounded by microglial cells. Microglia are endowed with P2X7Rs, whose stimulation by high concentrations of ATP causes the activation of intracelllular caspase-1 triggering thereby the apoptotic caspase cascade. P2X7R-mediated processes result in the release of inflammatory cytokines (e.g., IL-1β), proteases, reactive oxygen species (ROS) and the excitotoxic glutamate (Glu). During AD, hyperphosphorylated tau forms intracellular neurofibrillary tangles in neurons. All these damaging conditions lead to neuronal necrosis and microglial apoptosis. The binding of IL-1β to its receptors at neurons modifies long-term potentiation (LTP) and -depression (LTD), as well as synaptic transmission. Microglial activation may also cause excessive synaptic pruning by phagocytosis, being a neurodegenerative factor. Reproduced with permission from [145].

A consensus has emerged that Aβ promotes neuronal degeneration and that microglia are involved in this process [153,154,155] (Figure 2). Subsequently a relationship between Aβ deposition and P2X7Rs localized at activated microglia was observed [156,157]. Aβ triggered the increase in [Ca^2+^]_i_ of wt microglia grown in a culture preparation, and also facilitated the ATP release and IL-1β secretion from these cells by permeabilizing their plasma membrane [157]. However, none of these responses occurred when microglia were prepared from P2X7 deficient mice. Likewise, intra-hippocampal injection of Aβ caused a large accumulation of IL-1β in wt but not in P2X7^−/−^ mice. In accordance with these findings Aβ injection into the hippocampus of wt rats caused an increase in P2X7R expression, astrogliosis and loss of hippocampal neurons [158]. All effects were antagonized by BBG, which also prevented the inflammatory responses to Bz-ATP injection. P2X7R expression was associated with Aβ plaques and was localized at microglial cells [158].

The next question to be answered was whether microglia are sources of bioactive/inflammatory molecules, and if yes, by which mechanisms do they damage neurons under the influence of Aβ? In mixed neuron-microglia cultures, fibrillar Aβ (fAβ) aggregates stimulate microglia to produce superoxide by activating nicotinamide adenine dinucleotide phosphate (NADPH) oxidase [159] resulting in neurotoxicity [160]. Similarly, ATP and Bz-ATP were shown to induce superoxide production in microglial cultures through P2X7R activation [161,162]. Hence, fAβ was suggested to trigger ATP release which in turn activated NADPH oxidase via P2X7R occupation. The in vitro finding that ROS is stimulated in microglia to damage neurons [159] was also confirmed in a mouse model of AD [163]. Up-regulation of P2X7Rs and ROS production in microglia were paralleled with Aβ increase and correlated with injury of postsynaptic density 95-positive dendrites in the cerebral cortex of APP_swe_/PS1dE9 mice.

In an elegant approach, Tg(P2X7-EGFP) mice were crossed with the J20 hAPP mice which express a mutant form of the human amyloid protein precursor [164]. Thereby the distribution of the P2X7R message could be observed in a well-established Alzheimer mouse model. Neuroinflammation by Aβ peptide caused changes in P2X7R distribution pattern, with an increased expression in microglial cells. It was also shown that microglial cells migrated to concentrate around senile plaques.

In perfect agreement with the involvement of the pro-inflammatory receptor P2X7 in AD it was noticed that the 489C>T small nucleotide polymorphism (SNP; coding for His155Tyr) was significantly less frequent in AD patients than in healthy patients of matching age [165]. In addition, the presence of the 1513A<C allele (coding for Glu496Ala) in the absence of the 489C>T allele decreased the probability of having AD by about four-fold. This confirmed the tight correlation between Aβ and unmodified P2X7Rs in the onset and progression of AD.

Activation of P2X7Rs stimulated the release of the beneficial sAPPα from mouse neuroblastoma cells expressing human APP [166]. sAPPα shedding was inhibited by P2X7R antagonists or knockdown of P2X7Rs with siRNA and was not observed in neural cells from P2X7^−/−^ mice. These findings appeared to suggest an unexpected favorable effect of P2X7R agonists in AD, but as published later, was overridden by the Aβ peptide-mediated release of chemokines, particularly CCL3, which is associated with pathogenic CD8^+^ T cell recruitment during AD [35]. Another group of authors reported that P2X7Rs inhibited glycogen synthase kinase 3 (GSK-3) activity in two cellular lines, and J20 transgenic mice [167,168]. Blockade of P2X7Rs resulted thereby in a significant decrease in the number of hippocampal amyloid plaques.

Systemic administration of BBG diminished spatial memory impairment and cognitive deficits in the Morris water maze test of a mouse AD model produced by injecting soluble Aβ into the hippocampal CA1 region [169]. In addition, it was shown that Aβ-induced loss of filopodia and spine density in cultured hippocampal neurons was prevented by BBG. As a cellular model of memory, LTP in the hippocampal CA1 area was inhibited by soluble Aβ oligomers in a manner prevented by the NR2B antagonist ifenprodil [170]. This suggested that NMDA-Rs located at dendritic spines are involved in the depression of LTP [171]. Continued exposure of highly differentiated cultures of hippocampal neurons with Aβ resulted in abnormal spine morphology, with induction of long thin spines and a reduction in spine density. While CA1-LTP induced by high frequency stimulation was inhibited by Aβ, LTD induced by low-frequency stimulation in granule cells of the hippocampus was increased by the amyloid β-peptide [172].

The acute phase protein serum amyloid A, a putative TLR4/TLR2 agonist and the established TLR4R agonist LPS were used to prime purified microglial cultures prepared from the rat cortex [173]. Priming of microglia with serum amyloid A followed by the administration of ATP resulted in a robust release of IL-1β into the culture medium. The selective P2X7R antagonist A-740003 blocked the ATP-dependent release of IL-1β. Given that serum amyloid A is detected in the brain of AD patients, these results provide a further mechanism for the P2X7R-related microglial neurotoxicity during AD. As mentioned previously, PSN-1 and -2 are involved in the cleavage of APP. It was therefore interesting to observe that PSN-2 deficiency facilitated the Aβ-induced neuroinflammation and neuronal injury by up-regulating P2X7R expression in microglial cells of the hippocampal CA1, CA2 and CA3 regions [174]. This was accompanied by an increase of Aβ-induced ATP release and pro-inflammatory cytokine (IL-1β, TNF-α) release from microglia. Eventually, PSN-2^−/−^ mice demonstrated increased cognitive impairment in the Morris water maze-test apparently due to the developing cerebral injury.

## 8. Parkinson’s Disease (PD)

PD is clinically characterized by motor symptoms, including bradykinesia, rigidity, tremor, and postural instability. Hallmark pathological changes are intra-neuronal and intra-axonal α-synuclein positive inclusions (Lewy bodies and Lewy neurites) and loss of dopaminergic neurons in the substantia nigra pars compacta and their projection areas in the striatum [175,176]. The overwhelming majority of PD cases are idiopathic, and can be modeled in animal studies by exposure to toxins selectively destroying noradrenergic/dopaminergic neurons in the substantia nigra (6-OH-dopamine [6-OH-DA], MPTP) or treatment with environmental toxins/pesticides inhibiting the mitochondrial respiratory chain complex 1 (rotenone, paraquat) [177,178]. Few PD cases have a genetic etiology and may be investigated after knockout or transgenic modifications of PD-related genes (parkin, α-synuclein) in mice. More recently, aberrant functioning of the immune system has been proposed as a critical component of susceptibility to and progression of PD [175]. Activated microglia were found in the striatum and substantia nigra of patients with PD [179] and pro-inflammatory cytokines such as IL-1β and TNF-α were increased in the cerebrospinal fluid of such patients [180,181]. While originally these reactions were considered to be a consequence of the disease, nowadays they are supposed to be of etiological significance [182] and may be modeled in animals by the injection of LPS [178].

Rats were injected in their striatum unilaterally with 6-OH-DA to achieve a destruction of dopaminergic nerve terminals which than evolve after about a fortnight to a manifest dopaminergic cell loss in the substantia nigra and the emergence of motor disturbances [183]. BBG was applied i.p. 2 h after 6-OH-DA injection and then every 48 h for two weeks. This P2X7R antagonist attenuated all symptoms of hemiparkinsonism such as the increased contralateral rotations in the apomorphine test, the short-term memory impairment determined in the Morris water maze, the reduction of dopamine content in the striatum, and the striatal microgliosis/astrogliosis [183]. In support of these findings, 6-OH-DA treatment increased the P2X7R protein in the lesioned striatum, an effect reversed by BBG application [184]. Similarly, BBG also antagonized the hemiparkinsonian rotation behavior. In vitro autoradiography with [^11^C]JNJ-717 to label P2X7Rs and [^18^F]DPA-714 to label the microglial marker translocator protein demonstrated in rats receiving unilaterally 6-OH-DA, at day 14 post-injection a maximum P2X7R binding, in good temporal correlation with the maximum binding affinity of the microglial marker [185].

The protective effect of BBG treatment against 6-OH-DA-induced hemiparkinsonian symptoms including microglial activation in the substantia nigra [186] and striatum [187] was repeatedly confirmed. BBG attenuated the oxidative stress (lipid peroxidase and superoxide dismutase activities), as well as the deterioration of mitochondrial integrity (changes in mitochondrial membrane potential and mitochondrial complex-I, II, III and IV-activities) [187]. BBG also attenuated the mitochondrial-linked apoptosis as observed by a decrease in the expression of cytochrome-C, caspase-9 and caspase-3 in the striatum. After the application of ATP, cell swelling, loss of endoplasmic reticulum integrity, the formation of large cytoplasmic vacuoles and subsequent cytolysis occurred in the P2X7R bearing SN4741 dopaminergic cell line [188]. However, caspase inhibitors had no beneficial effect, indicating that necrosis rather than apoptosis is associated with ATP-induced cell death.

The microglial cell line BV2 and primary cultured microglia responded to α-synuclein with NADPH oxidase activation [189]. The activation of NADPH oxidase depended on the presence of P2X7Rs and has led to the cleavage of caspase-3 in the dopaminergic cell line SH-SY5Y [190]. The oxidative damage by α-synuclein of SH-SY5Y cells was due to P2X7R activation and the simultaneously occurring increased release of ATP as well as its decreased degradation by ecto-ATPase. α-Synuclein aggregates in addition caused the release of the excitotoxic glutamate from cultured microglial cells [191]. Thus, α-synuclein, a constituent of Lewy bodies present during PD may activate microglia which then damage dopaminergic neurons on the one hand by the secretion of ATP/glutamate and on the other hand by the release of ROS.

The injection of LPS into the substantia nigra revealed the enhanced expression of P2X7Rs on microglia and the consequent loss of dopaminergic neurons [192]. Treatment with BBG reduced both the activation of microglia and the loss of nigral dopaminergic neurons. It was concluded that the activation of microglia by LPS and the P2X7R-dependent synthesis of pro-inflammatory cytokines is the reason for the death of substantia nigra neurons [193] supposed to be responsible for the clinical symptoms of PD.

## 9. Multiple Sclerosis (MS)

MS is based on an autoimmune inflammatory reaction to myelin components leading to oligodendrocyte death and axonal demyelination/damage in the CNS [194,195,196]. Axonal demyelination causes failure of action potential propagation and short circuiting of action potentials between neighboring axons. The symptoms of the disease are visual disturbances, motor impairments, fatigue, pain, and cognitive deficits. Clinically three major types of MS can be defined: (1) Relapsing/remitting MS characterized by unpredictable relapses followed by remissions with no signs of disease activity; (2) Primary progressive MS with no remissions after the initial symptoms, and; (3) Secondary progressive MS occurring in most patients by a transition of relapsing/remitting MS into this state, characterized by no relapse phases at all.

The relapse/remitting type of MS is characterized by blood brain barrier (BBB) damage and prominent infiltration of autoreactive T cells together with activated B cells as well as monocytes, dendritic cells and natural killer T cells that develop an aberrant destructive response against myelin antigens. With the progression of the disease as well as in primary progressive MS, the infiltration of peripheral immune cells through the BBB declines and the inflammatory reaction is maintained by microglia and astrocytes [194,195,196,197]. A generally accepted animal model of MS is experimental autoimmune encephalomyelitis (EAE) induced with the injection of synthetic myelin oligodendrocyte glycoprotein peptide or homogenates of spinal cord tissue combined with the application of an inflammatory compound (pertussis toxin, CFA, Mycobacterium tuberculosis, etc.).

Of the P2XR family, P2X4Rs were found to be intimately involved in MS, improving remyelination [198,199]. By contrast, P2X7Rs appear to be deleterious during autoimmune diseases in general and MS in specific [9,195,196]. In post-mortem human spinal cord specimens of MS patients, a higher density of P2X7-IR microglial cells was observed than in their non-MS counterparts [200,201]. Enhanced P2X7R-IR at morphologically activated microglia was observed already a few days after antigenic injection to induce EAE in rats [202]. BBG delayed the onset of EAE and partially inhibited the development of neurological symptoms. BBG also suppressed the level of the pro-inflammatory cytokines, IL-1β, IL-6, and TNF-α. Moreover, P2X7^−/−^ mice were found to be more resistant to EAE than wt mice exhibiting less neuroinflammation and axonal damage [203].

In addition to microglia, astrocytes also become activated in an early phase of EAE and express increased P2X7R-IR [204]. BBG decreased astrogliosis in the forebrain of immunized rats and alleviated the neurological symptoms. In view of the reported preferential localization of Panx-1 channels in astrocytes and their confirmed role in the release of the potentially neurotoxic ATP [145], it was interesting to observe that Panx-1 KO mice displayed a delayed onset of clinical signs of EAE and decreased mortality compared to wt mice [205].

Eventually, we arrive at the target cells of the MS injury, the oligodendrocytes, forming the myelin sheath of axons in the CNS. The considerable Ca^2+^ permeability of P2X7Rs and the subsequent overload of the cell interior by Ca^2+^ were supposed to induce exocytotic death of oligodendrocytes [206,207]. In this manner ATP, in contrast to another excytotoxin, glutamate stimulating NMDA-Rs, acted already at the resting membrane potential and desensitization of the receptor did not prematurely terminate the agonist effect. ATP and Bz-ATP caused inward transmembrane currents in cultured oligodendrocytes which were inhibited by oxATP and BBG; similarly, ATP/Bz-ATP triggered a massive influx of Ca^2+^ into these cultured cells [207]. Further, ATP or Bz-ATP killed oligodendrocytes in vitro (cell cultures), in situ (perfused optic nerves), or in vivo (infusion onto optic nerves by osmotic minipumps). BBG also ameliorated the motor deficits observed in EAE mice in contrast to vehicle-treated animals. Eventually, the number of P2X7R-IR oligodendrocytes increased in human optic nerve samples obtained by autopsy of MS patients in comparison with those obtained from control non-MS patients.

A hallmark of MS is the infiltration of peripheral immune cells through the permeable BBB into the CNS. P2X7R-IR was identified at the abluminal surface of brain microvessels and was co-expressed with the platelet-derived growth factor β receptor (PDGFβR), a marker of pericytes [202]; pericytes are involved together with endothelial cells in BBB formation. During the course of EAE, the protein levels of PDGFβR and the tight junction protein claudin-5 decreased. Administration of BBG to immunized rats reduced the clinical signs of EAE and enhanced the previously decreased protein expression of both PDGFβR and claudin-5. Hence, EAE damaged the BBB, an effect readily antagonized by intravenous (i.v.) BBG.

In contrast to the well founded and repeatedly confirmed hypothesis that P2X7R activation is cell damaging, exacerbation of EAE was reported in P2X7 KO mice to take place due to the loss of apoptotic activity in lymphocytes. Chen et al. [208] found that in the course of EAE a direct damage by ATP to P2X7R-possessing oligodendrocytes is counterbalanced by the loss of apoptosis in a lymphocytic cell population. Reduced interferon-γ (IFN-γ) and NO levels were detected in the CNS tissue of P2X7^−/−^ mice, as well as less TUNEL staining (a marker of apoptosis) of inflamed CNS tissue. However, as mentioned already above, another group of researchers found that the balance between CNS damage caused by infiltrating T lymphocytes and intrinsic astrocytes/microglia resulted in a reduction of the EAE disease intensity in P2X7 KO mice [203].

Not only infiltrating T lymphocytes but also infiltrating monocytes are deleterious in the CNS. It was shown that MS increased the shedding of extracellular vesicles (supposedly containing IL-1β), and treatment of MS patients reduced monocyte-derived vesicle production [209]. The suggestion that infiltrating peripheral blood cells lose their significance as damaging factors in the later phases of MS, were supplied by the decrease of P2X7R-IR on monocytes and up-regulation on astrocytes to contribute to pertinent inflammation in MS [201].

MS is not considered to be a hereditary disease; however, a number of genetic variations, among others, SNPs of the P2X7R, have been shown to decrease or increase the risk for this illness. The rs28360457 SNP which codes for the Arg307Gln variant of P2X7R exhibits loss-of-function, as confirmed by measurement of ethidium bromide uptake into monocytes [210]. It is located in the binding site of the receptor [211] and is associated with protection against MS risk [210]. It always occurs together with rs7958311 (Arg270His; extracellular loop) suggesting a single 307Gln-270His haplotype that confers dominant negative effects on P2X7R function and lower risk for MS. The T allele of the rs17525809 polymorphism which yields Val76Ala (extracellular loop), shows a gain-of-function, consisting of larger electrophysiological responses and higher ethidium bromide uptake [212]. This SNP is associated with a more frequent occurrence of MS. In this case a haplotype with rs208294 (His155Tyr; extracellular loop) was observed.

## 10. Amyotrophic Lateral Sclerosis (ALS)

ALS causes the death of neurons in the brainstem and spinal cord controlling voluntary movements [213,214,215]. This disease can affect people of any age, but usually starts around the age of 60 and in inherited cases around the age of 50. The average survival from onset to death is two to four years, and most people die because of respiratory failure. In a most commonly used animal model of ALS, mice overexpress multiple copies of a human variant of superoxide dismutase 1 (SOD1-G93A) and develop clinical signs of ALS at 70–140 days of age [216]. Multiple pathophysiological mechanisms develop in ALS such as oxidative stress, neuroinflammation, mitochondrial dysfunction, protein misfolding and aggregation, lack of trophic growth factors, aberrant axonal conduction, and BBB impairment [215].

An upregulation of P2X7R-IR and protein in human postmortem spinal cord specimens of ALS patients was observed and assigned in the first line to microglia/macrophages in affected regions [200]. Accordingly, in cultured spinal cord microglia prepared from SOD1-G93A mice, the stimulation of P2X7Rs by Bz-ATP enhanced the microglial content of TNF-α and cyclooxygenase-2, causing a toxic effect on two neuronal cell lines [217]. All these changes were prevented by the antagonist BBG. In SOD1-G93A microglia, the activity of the main ROS producing enzyme, NADPH oxidase-2 was increased by Bz-ATP [218]. This microglia-mediated neuro-damaging mechanism was prevented by knocking out the P2X7R and by the use of BBG. In addition to SOD1-G93A microglia producing neurotoxic ROS, astrocytes taken from these animals were also found to participate in the P2X7R-induced ROS production [219]. Treatment with the superoxide scavenger MnTBAB, the P2X7R antagonist BBG, and pre-incubation with apyrase to degrade endogenous extracellular ATP, all procedures prevented the death of motoneurons, co-cultured with SOD1-G93A astrocytes. Hence, P2X7R activation in spinal cord microglia and astrocytes initiated a neurotoxic phenotype that leads to motoneuron death.

In the following experiments, both aggregated and soluble SOD-1-G93A activated the NLRP3 inflammasome in primary mouse microglial cultures leading to the release of IL-1β [220]. Importantly, SOD1-G93A was unable to induce IL-1β secretion from microglia genetically deficient in NLRP3. These data demonstrate that ALS microglia express NLRP3 and that the pathological ALS protein activates the microglial NLRP3 inflammasome to generate ROS and ATP as key elements of neurotoxicity.

In contrast to the above demonstrated beneficial effect of P2X7R ablation, this procedure was found to anticipate the clinical onset and significantly worsen the disease progression in SOD1-G93A mice [221]. There was also increased astrogliosis, microgliosis, motoneuron loss and induction of the pro-inflammatory marker NADPH oxidase-2. In an attempt to explain this controversy, BBG was applied to SOD-G93A mice in the late pre-symptomatic phase of the disease [222]. It was found that BBG enhanced motor neuron survival and reduced microgliosis in the lumbar spinal cord, reducing inflammatory markers, such as NADPH-2 oxidase, IL-1β, and BDNF. Hence, P2X7R activation may be favorable in the early asymptomatic phase of disease development, but later it is unequivocally deleterious.

Another group of researchers found only a mild effect of P2X7R antagonism in female SOD1-GH93A mice by BBG [223] and no effect at all by JNJ-47965567 a BBB permeable highly selective P2X7R antagonist [216]. The conclusion was that the effect of P2X7R blockade depends on the type of antagonist used, its dose regimen, the mode of application, the clinical phase of the disease, and the gender of the mice [224]. This makes it rather unlikely that this treatment will be ever introduced into clinical practice.

## 11. P2X7 Receptor Antagonists as Possible Pharmacological Tools to Treat Neurodegenerative Diseases

It has to be pointed out that my aim was to begin the journey around neurodegenerative diseases with the P2X7Rs involved rather than with the ligands used for their characterization. A couple of years ago the molecular, biophysical, functional and cellular properties of all recombinant homomeric P2XRs including those of P2X7Rs were summarized in a IUPHAR-initiated paper, and reference was made to the agonists/antagonists available at this time [225]. Of course, more recently the developments were impressive; I apologize to the reader for neglecting the medicinal chemistry and detailed pharmacodynamic/-pharmacokinetic aspects of P2X7R antagonists and refer in this respect to excellent review articles of other authors, especially of the Janssen group headed by Anindya Bhattacharya [29,226,227,228,229].

P2X7R antagonists block the release of inflammatory cytokines from peripheral macrophages and have been intensively investigated for their therapeutic effects in rheumatoid arthritis [230,231] and Crohn’s disease [232]. Whereas these antagonists against expectations did not produce a beneficial effect in the case of rheumatoid arthritis, they improved symptoms in patients with moderate-to-severe Crohn’s disease. Nonetheless, the development of P2X7R antagonists for peripheral therapeutic indications was terminated by various pharmacological companies carrying on these studies [33,228].

Mechanical damage to the CNS, stroke/ischemia, neuropathic pain, as well as AD, PD, MS, or ALS lead to irreversible damage of the neural tissue by disease-specific causative factors; however, in the neighborhood of the immediately afflicted area secondary neuroinflammation causes additional injury. Because of the intimate involvement of P2X7Rs in neuroinflammatory processes, antagonists to this receptor are supposed to beneficially modulate the secondary consequences of the enlisted diseases. It is essential for the P2X7R antagonists used that they should freely pass the BBB on systemic application and have sufficient receptor occupancy in the CNS of experimental animals and human beings.

A frequently used antagonist in various in vivo systems is BBG which is considered to pass the BBB quite avidly. BBG has been shown to selectively block recombinant P2X7Rs expressed in HEK293 cells in the nanomolar range [233]. In spite of the excellent selectivity of BBG for P2X7Rs when compared with that for P2X1, P2X2, P2X3, P2X2/3, P2X4, and P2X1/5Rs [233], it was also shown to inhibit murine neuronal voltage-gated sodium channels in similar concentrations which block murine P2X7Rs [234].

Somewhat later it was reported that intravenously applied BBG reduced spinal cord anatomical damage and improved motor damage 4 days after weight drop injury to the spinal cord [60]. It was also stated that BBG is a derivative of the commonly used blue food color (FD6C blue No. 1), which crosses the BBB. However, at a single i.v. dosage of 50 mg/kg, BBG may preferentially pass the leaky blood-spinal cord barrier rather the intact one [227]. This finding forced a whole range of authors to use repeated systemic applications of BBG to achieve reliable improvement of functional limitations encompassing neurodegenerative diseases (see the present review article). However, a much better choice than BBG are more recently developed P2X7R antagonists with brain/plasma distribution ratios of ~1 (e.g., JNJ-54175446 and JNJ-55308942) indicating very good permeability through the BBB [235,236]. Both compounds have a 1,2,4-triazolopyrazine core revealing strong enantiomeric preference for the P2X7R in rat and human CNS tissues [226]. These Janssen compounds approximately equal the compounds of Pfizer (compound 7f) and Abbot (A-438079) in their brain/plasma distribution ratios [29] and have the advantage that at least JNJ-54173717 is also available as a [^11^C]-labeled PET ligand [237]. Further JNJ compounds of different structure were also developed as radioligands or PET ligands to enable the measurement of binding site specificity [238] and quantification of P2X7R expression in the human brain [239].

## 12. Transgenic P2X7 Mice as Experimental Tools for the Investigation of P2X7R Participation in Neurodegenerative Diseases

In addition to the use of pharmacological antagonists, P2X7^−/−^ mice were also routinely utilized in a search for P2X7R mediated effects in rodents. It is imperative to be cautious with the ‘standard’ P2X7 KO mice generated a couple of years ago in the pharmacological companies Glaxo and Pfizer. In the hippocampus of the two supposedly P2X7R-deficient mice strains, there was unaltered immunohistochemical staining with the respective antibodies when compared with wt animals [54]. By contrast, the receptor protein was missing in the peripheral organs of submandibular gland and lung of P2X7^−/−^ mice. An explanation for this finding was supplied by the discovery of immunologically and also functionally active splice variants of the receptor which are not inactivated by the two KO strategies. P2X7R-K (see Section 2), in which the entire N-terminus and the first half of transmembrane domain 1 are replaced, is functional and in case of the Glaxo KO mouse [54] escapes the KO strategy [17,240]. Likewise, use of two alternative exons 13 (13b and 13c) has been described [241] and the resulting proteins contain considerably shorter C-termini. Transcript 13b escapes the KO strategy used in the Pfizer KO mouse [242].

Another method utilized to identify P2X7Rs are Tg(P2X7-EGFP) mice in which EGFP is under the control of the P2X7 promoter and can be identified by observing the immunohistochemical staining under a fluorescent microscope [88,96] (see Section 5). It was criticized that BAC transgenic mice do not reliably differentiate between P2X7 and P2X4R involvement [97]. However, more recently another BAC transgenic mouse was generated; a comparative analysis of the P2X7-EGFP transgene expression and endogenous P2X7 expression in wt mice using a novel P2X7-specific nanobody-rblgG fusion construct confirmed identical cell-type specific distribution [32].

Eventually, a conditional humanized mouse was generated in which the P2X7 allele is accessible to spatially and temporally controlled Cre recombinase-mediated inactivation [243]. In contrast to previously generated KO mice, none of the described P2X7 splice variants evaded this null allele. However, the experimental evidence for the validation of this mice is relatively limited and it has to be mentioned that the strong expression of the mRNA level for P2X7 in the wt hippocampal CA3 area [243] is not reflected by EGFP expression in the Tg(P2X7-EGFP) mouse line (compare: http://www.gensat.org/).

In view of these uncertainties inherent in the presently available knockout/knockin and transgenic mouse models it is strongly advisable to use selective P2X7R antagonists in parallel to any investigation taking advantage of such genetically modified mice.

## 13. Conclusions

Neurodegenerative diseases of the CNS acquire increasing significance in industrialized countries in parallel with the continuous prolongation of the life duration of their aging population. A causal therapy is not known for late consequences of mechanical damage, stroke/ischemia, neuropathic pain, as well as AD, PD, MS, or ALS. Therefore, it is of eminent significance to look for possibilities to at least slow down the progredience of the pathological processes. This is feasible by the use of BBB permeable P2X7R antagonists with excellent bioavailability, which fortunately we have at our disposal. The basis of this therapeutic manipulation lies in the P2X7R-initiated microglia/neuroglia-mediated damage inflicted upon healthy neural tissue in the course of neurodegenerative diseases. Thus, I strongly believe that P2X7R antagonistic drugs with good bioavailability, excellent penetration into the CNS, and a low incidence of unwanted effects are potential drugs for the symptomatic treatment of neurodegenerative diseases. Pharmacological companies should be alerted to enter clinical trials—even when tedious, because of the very slow progredience of these diseases—with the already developed and pre-clinically tested compounds.

## Abbreviations

AD	Alzheimer’s disease
ALS	amyotrophic lateral sclerosis
BBB	blood brain barrier
BBG	Brilliant Blue G
Bz-ATP	Dibenzoyl-ATP
DAMPs	danger-associated molecular patterns
EGFP	enhanced green fluorescence protein
GFAP	glial fibrillary acidic protein
i.c.v.	intracerebroventricular
IL-1β	interleukin-1β
IR	immunoreactivity
KO	knockout
LPS	lipopolysaccharide
MCAO	medial cerebral artery occlusion
MS	multiple sclerosis
NADPH	nicotinamide adenine dinucleotide phosphate
NLRP3	nucleotide-binding leucine-rich repeat pyrin domain containing 3
NTPDases	ecto-nucleoside triphosphate diphosphohydrolases
OGD	oxygen-glucose deficiency
oxATP	oxidized ATP
PAMPs	pathogen-associated molecules
Panx-1	pannexin-1
PD	Parkinson’s disease
R	receptor
ROS	reactive oxygen species
SE	status epilepticus
SOD-1	superoxide dismutase 1
Tg	transgenic
TLR	toll-like receptor
TNF-α	tumor necrosis factor-α
TUNEL	terminal deoxynucleotidyl transferase dUTP nick end labeling
α,β-meATP	α,β-methylene ATP
